# Computing the Work of Solid–Liquid Adhesion in Systems with Damped Coulomb Interactions via Molecular Dynamics: Approaches and Insights

**DOI:** 10.1021/acs.jpca.2c03934

**Published:** 2022-08-05

**Authors:** Donatas Surblys, Florian Müller-Plathe, Taku Ohara

**Affiliations:** † Institute of Fluid Science, Tohoku University, 2-1-1 Katahira, Aoba-ku, Sendai, 980-8577, Japan; ‡ Eduard-Zintl-Institut für Anorganische und Physikalische Chemie, Technische Universität Darmstadt, D-64287, Germany

## Abstract

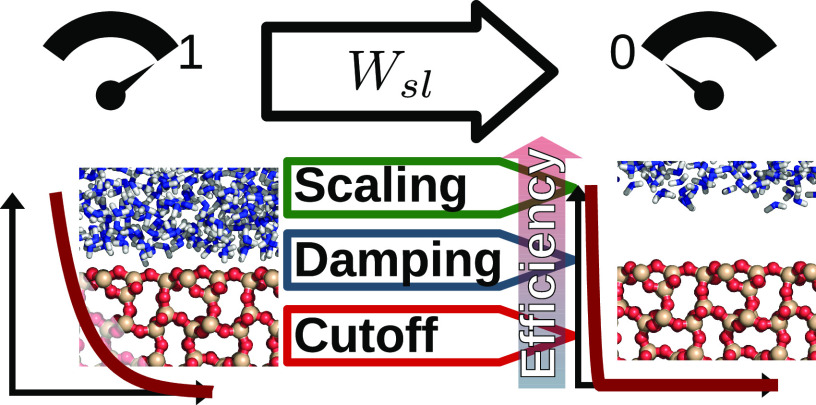

Recently, the dry-surface method [Langmuir2016, 31, 8335−8345] has been developed to compute the work of adhesion of solid–liquid and other interfaces using molecular dynamics via thermodynamic integration. Unfortunately, when long-range Coulombic interactions are present in the interface, a special treatment is required, such as solving additional Poisson equations, which is usually not implemented in generic molecular dynamics software, or as fixing some groups of atoms in place, which is undesirable most of the time. In this work, we replace the long-range Coulombic interactions with damped Coulomb interactions, and explore several thermal integration paths. We demonstrate that regardless of the integration path, the same work of adhesion values are obtained as long as the path is reversible, but the numerical efficiency differs vastly. Simple scaling of the interactions is most efficient, requiring as little as 8 sampling points, followed by changing the Coulomb damping parameter, while modifying the Coulomb interaction cutoff length performs worst. We also demonstrate that switching long-range Coulombic interactions to damped ones results in a higher work of adhesion by about 10 mJ/m^2^ because of slightly different liquid molecule orientation at the solid–liquid interface, and this value is mostly unchanged for surfaces with substantially different Coulombic interactions at the solid–liquid interface. Finally, even though it is possible to split the work of adhesion into van der Waals and Coulomb components, it is known that the specific per-component values are highly dependent on the integration path. We obtain an extreme case, which demonstrates that caution should be taken even when restricting to qualitative comparison.

## Introduction

The miniaturization of semiconductors continues to advance with the latest lithography technology enabling resolutions below 10 nm.^1^ At such scales, the surface-to-volume ratio greatly increases and interfacial effects start to have a great influence on overall properties, such as heat dissipation,^2,3^ wetting,^4−6^ and flow.^7,8^ For all these topics, molecular dynamics (MD) simulation has been widely and successfully used for several decades,^9−12^ either as a complement or as an alternative to experimental work.

This Article focuses on using MD to obtain the solid–liquid work of adhesion, that is, the reversible work needed to separate the solid and liquid phases. The solid–liquid work of adhesion *W*_sl_ is an important interface property and is related to wettability via the Young–Dupré equation:
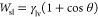
1where γ_lv_ is the liquid–vapor interfacial tension and θ is the contact angle. In addition, a correlation between wettability, therefore, also work of adhesion, and interfacial thermal properties has also been reported.^13−17^ The work of adhesion, or the interfacial tension, has often been obtained from the contact angle of simulated droplets^10^ and eq 1. While straightforward, easy to implement and demonstrated to strictly hold for smooth surfaces,^18^ complications such as ambiguity of fitting parameters, long relaxation times,^19^ pinning on nonhomogeneous surfaces^20,21^ and size effects such as line tension^22−25^ are often encountered creating uncertainty in the results. As an alternative and less computationally intensive method, the application of the Wilhelmy equation via MD has also been proposed but is yet to be applied to more complex systems.^26^

Recently, obtaining the work of adhesion directly via thermodynamic integration has become more prominent. Various integration paths have been taken, such as using a virtual piston to separate the solid and liquid phases (phantom-wall method),^27^ switching solid–liquid interactions to repulsive-only (dry-surface method)^28^ or turning the liquid phase into an ideal gas.^29^ In particular, the dry-surface method is very straightforward, has the advantage that the systems midpath can also be physical and of interest, and has been applied not only to atomistic systems,^18,21,30,31^ but also coarse-grained ones.^32,33^ The basic concept is that both van der Waals and Coulomb interactions between solid and liquid are gradually switched off via coupling parameters. The work of adhesion is obtained from partial derivatives of the system Hamiltonian with respect to the coupling parameters. Note that the dry-surface method obtains the reversible work needed to separate the solid–liquid interface into solid-vacuum and liquid-vacuum interfaces, while contact angles are usually measured in humid environment, and in such case eq 1 would not strictly hold. In practice, however, there is little effect for a large number of systems, where liquids with very low saturated vapor pressure, such as water are used.^34^ Indeed, a good match between the contact angle of water on various silica surfaces and the work of adhesion obtained via an equivalent method to the dry-surface approach has been obtained in a previous numerical computational work by one of the authors.^35^ In case of liquids with significantly higher vapor pressure, it has been demonstrated that there is a non-negligible discrepancy between solid–vacuum and solid–vapor interfacial free energies that can result in overestimation of the solid–liquid work of adhesion by over 10%, although it can be corrected by also obtaining the work of solid–vapor adhesion via the dry-surface method.^18,26^ A similar approach has also been successfully used to predict contact angles of fluid mixtures above the fluid saturation pressures via the phantom-wall method.^36^

Most realistic systems have Coulombic interactions at the interfaces, and the authors have demonstrated that it is possible to use the dry-surface method also for systems with long-range Coulombic solid–liquid interactions.^37^ Unfortunately, while theoretically straightforward, this either requires solving additional Poisson equations, which is generally not implemented in generic MD software, or fixing some groups of atoms in place, which in itself changes interfacial properties and is often undesirable. To get around this practical problem, we replace in this work the long-range Coulombic interactions with the widely used damped interactions developed by Fennell et al.,^38^ which is an improvement on the Wolf model^39^ and ensures smooth decay of both energy and force at the cutoff. This allows several possible approaches for turning off solid–liquid Coulombic interactions, and makes the implementation in generic MD software easier. We investigate six possible thermodynamic integration paths and demonstrate a suitable scheme to apply the dry-surface method for obtaining the solid–liquid work of adhesion. At the same time, we also provide several more general insights into effect of using damped Coulombic interactions on the interfacial properties, and demonstrated the difficulty of dividing the work of adhesion into nonarbitrary physically meaningful contributions.

## Simulation and Analysis Methods

The overall potential and simulation system models are based on the silica–water system of our previous work.^37^ A detailed description will be given only for conditions that are different in this work, while identical conditions will be described in brief.

### Potential Model

The flexible SPC/Fw model^40^ was used for water molecules. An α-cristobalite (101̅) face was used for the silica surface, with the potential parameters taken from the work of Emami et al.^41^ Unlike in our previous work, solid atoms were not constrained, and bonds and angles in both water and silica surface models were maintained by their respective harmonic potentials.

Intermolecular van der Waals interactions between solid and liquid atoms were expressed via the 12–6 Lennard-Jones (L-J) potential:
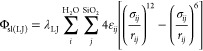
2where *r*_*ij*_ is the distance between the atoms, while σ and ε are the L-J length and energy parameters. The L-J parameters between two different atom types were obtained via the Lorentz–Berthelot mixing rules: σ_*ij*_ = 0.5(σ_*i*_ + σ_*j*_), . The λ_LJ_ is the coupling parameter used to modify the solid–liquid van der Waals interactions during thermodynamic integration. Solid–solid and liquid–liquid interactions were identical with eq 2, except there was no coupling parameter, that is, λ_LJ_ was always 1.

Intermolecular solid–liquid Coulomb interactions were represented via a short-range damped interactions modeled by Fennell:^38^
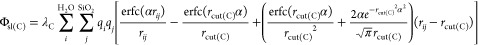
3where *q* is the charge of the corresponding atom, α is the damping parameter, and *r*_cut(C)_ is the cutoff distance of the Coulomb interactions. The damping parameter was set to a standard value of α = 0.2 Å^–1^.^38^ Similar to eq 2, the coupling parameter λ_C_ was used to turn off solid–liquid Coulombic interactions, while for solid–solid and liquid–liquid interactions it was always 1. As an alternative to using λ_C_ to turn-off solid–liquid Coulomb interactions, *r*_cut(C)_ or α were also used to obtain similar results.

While in our previous work the switching function from the CHARMM force field^42^ was used for the L-J interactions, and the PPPM method^43^ was used to treat long-range electrostatics; in this work, both eqs 2, 3 were used with a simple cutoff scheme at *r*_cut(LJ)_ = *r*_cut(C)_ = 12 Å for most computations. Thus, the Coulomb interactions are cut off at 2.4 times the decay length α^–1^ of the damped Coulomb potential. Only in several control systems full long-range Coulomb interactions were realized via the PPPM method with a precision of 10^–6^ instead of using eq 3.^43^ Regarding the treatment of L-J interactions, it has been demonstrated that the contact angle, that is, the work of solid–liquid adhesion, is also sensitive to the L-J cutoff length,^44^ where best agreement with experimental measurements was achieved by treating the L-J dispersive term as long-range, equivalent to that of Coulomb. Indeed, many force fields tend to underestimate surface tensions, and considering long-range dispersion forces often brings those values closer to the experimental ones,^45^ which creates a predicament as most force fields were optimized under an assumption of short-range L-J interactions. In recent years, several MD software packages have added long-range L-J solvers, and because of the existence of mixing rules, it is trivial to only modify a specific subset of interactions; therefore, no special treatment is needed when applying the dry-surface approach to such systems, with the only disadvantage being the increased computational cost.

Instead of having a gas phase to keep the system at saturated vapor pressure, a virtual wall was used as a piston to keep the system pressure at a specific value. The piston-liquid interaction was set as the 9–3 L-J potential acting only between the virtual piston and the oxygen atoms of water:
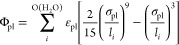
4where *l*_*i*_ is the distance of oxygen atom *i* from the virtual piston. The L-J parameters of σ_pl_ and ε_pl_ were setup to mimic the oxygen–silica interaction:
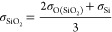
5
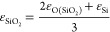
6
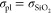
7
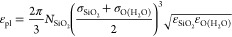
8where σ_SiO_2__ and ε_SiO_2__ were set as a rough approximation of the average L-J parameters for silica, and *N*_SiO_2__ ≈ 0.07 Å^–3^ is the atom number density of silica obtained from the density of 2.32 g/cm^3^ in the work of Emami et al.^41^ Strictly speaking, according to the mixing rules in this work, the value of σ_pl_ in eq 7 should have been set to 0.5(σ_SiO_2__ + σ_O(H_2_O)_), but the values are close and there is no real fundamental need for the piston–liquid interactions to have any specific parameters. The cutoff distance for piston–liquid interactions was set to *r*_cut(pl)_ = (2.5)^−1/6^σ_pl_ to have only repulsive interaction.

In addition, several control systems were created, where silica was replaced with a magnesium oxide (MgO) surface. The MgO potential parameters for L-J and Coulomb interactions were taken from the ClayFF force field,^46^ while the piston settings were kept unchanged.

### Simulation Systems

**Figure 1:**
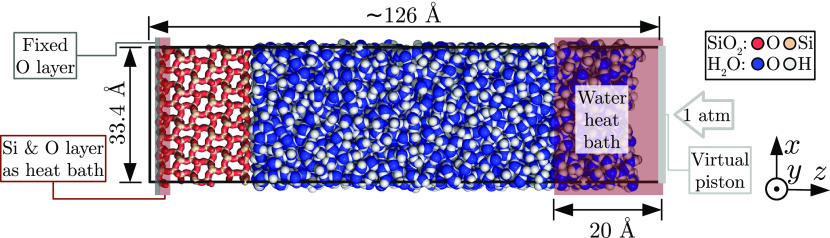
Front view of the simulation system. The depth (*y*) dimension is 34.8 Å.

A silica–water system displayed in Figure 1 was used, where there were no hydroxyl groups at the silica surface, that is, the surface was terminated by siloxane bridges. This is very similar to our previous work,^37^ except that no recalculation of silica unit cell parameters was done, and the silica surface models provided by Emami et al. were used,^41^ resulting in a system *xy* cross-section of approximately 33.4 × 34.8 Å^2^. Also, while in the previous work the whole silica surface was fixed, only the leftmost layer of 84 oxygen atoms was fixed in this work, as indicated in Figure 1. The number of water molecules was set to 7200, and was chosen to give a liquid layer that is large enough to have a bulk region not affected by short-range forces from both the solid surface and heat bath region, that is at least 50 Å thick.

An external pressure of 1 atm was applied to the virtual piston at the right side of Figure 1 by a constant external force of about 0.017 kcal/(mol Å) in the −*z* direction, and its position adhered to classical Newtonian equation of motion, where the piston mass was approximately 1163 kg/mol, obtained by setting the piston area density to 1000 g/(mol Å).

To damp the oscillation of the virtual piston, which occurred because of the repulsive-only interactions with water molecules, a Langevin thermostat,^47^ with a damping coefficient of 1000 fs and a control temperature of 0 K was coupled to the piston. To compensate for the energy loss due to damping and to control the system temperature, two additional Langevin heat baths were setup, both having the damping coefficient set to 100 fs and control temperature to 300 K. The first heat bath was coupled to the water molecules within 20 Å from the virtual piston. The second heat bath was set to the leftmost silicon and second-leftmost oxygen atom layers, composed of 84 and 168 atoms, respectively. Both are illustrated in Figure 1.

Several additional simulation system types were also constructed to better illustrate the effect of Coulomb damping versus full long-range electrostatics. First, silica–water systems were created with long-range electrostatic interactions, that is, no damping, where all silica atoms were fixed at their mean positions, with the other conditions being identical. The mean positions were obtained from the 8 ns sampling data of the initial state of the thermodynamic integration, that is, a system with normal silica–water interactions.

In addition, systems where MgO surface replaces silica were also created, illustrated in Figure S1, with either damped or full, long-range Coulomb interactions. The positions of the MgO atoms were fixed to form a perfect fcc (100) face, with a lattice constant of 4.212 Å. The system size was adjusted to be close to that of water–silica systems, resulting in a *xy* cross-section of approximately 33.7 × 33.7 Å^2^ with 12 MgO layers in the *z* direction. The number of water molecules was 10272.

Each system was equilibrated with various coupling parameters for at least 2 ns and either the following silica–water data of 8 ns or MgO–water runs of 14 ns were used for thermodynamic integration. The number of sampling points depended on the integration path and desired precision and will be discussed in detail below.

### Thermodynamic Integration

A detailed description of the dry-surface method is provided in the original paper,^28^ but in brief, it uses coupling parameters to reversibly switch the solid–liquid interactions from realistic to mostly repulsive, and it obtains the work of solid–liquid adhesion via thermodynamic integration. As in our previous work,^37^ we chose a reversible path, where λ = 0 indicates fully repulsive interactions, while λ = 1 represents the original systems for which we want to obtain the solid–liquid work of adhesion. Therefore, the work of adhesion can be obtained via
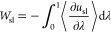
9where λ is an arbitrary coupling parameter in the range of 0 ≤ λ ≤ 1 and *u*_sl_ = Φ_sl_/*A* is the total solid–liquid potential energy per unit area, *A* being the area of the *xy* cross-section.

In case of our silica–water system in Figure 1, there are both van der Waals and Coulomb interactions between solid and liquid. As one simple approach, we chose to first turn off the Coulomb interactions and then gradually switch van der Waals interactions to repulsive via modifying the solid–liquid L-J interactions. For the Coulomb interactions, we tried three approaches: change the scaling coupling parameter in the range of 0 ≤ λ_C_ ≤ 1 (Coulomb scaling integration path; λ_C_ path), change the solid–liquid Coulomb interaction cutoff *r*_cut(C)_ in the range of 0 Å ≤ *r*_cut(C)_ ≤ 12 Å (Coulomb cutoff integration path; *r*_cut(C)_ path) and change the damping parameter α in the range of 0.2 Å^–1^ ≤ α ≤ 3.4 Å^–1^, where 3.4 Å^–1^ is a large enough value to effectively turn off Coulombic interactions (Coulomb damping integration path, α path). In accordance with eq 9, the contributions to the work of solid–liquid adhesion can be each obtained for these integration paths
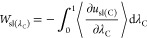
10
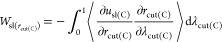
11
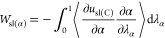
12where λ_cut(C)_ = *r*_cut(C)_/12 Å is used for eq 11, λ_α_= (3.4 Å^–1^ – α)/3.2 Å^–1^ is used for eq 12 and *u*_sl(C)_ is the Coulomb component of the solid–liquid energy per unit area. In case of van der Waals interactions, decreasing the coupling parameter to a very small value λ_LJ_ ≪ 1 is a straightforward way also used in previous works^28,37^ to create a mostly repulsive interaction. In this work, the values were taken in the range of 0^+^ < λ_LJ_ ≤ 1 (L-J integration path; λ_LJ_ path), where the “+” superscript indicates a tiny value larger than 0, as we only need to make the L-J potential well small enough to cause repulsive-only interaction, and setting it to 0 would be either numerically unstable or cause nonreversible change in the system. As the exact choice of the value has less effect than numerical uncertainty, we chose an extrapolation scheme to 0, that will be described in the integration scheme section, and use 0 notation in definite integral for simplicity. Note that there were no Coulombic contributions in solid–liquid interactions at this phase. The contribution to the work of solid–liquid adhesion can be obtained in a straightforward manner
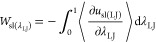
13where *u*_*sl*(LJ)_ is the L-J component of the solid–liquid energy per unit area.

We also adopted alternative integration paths where we modified both van der Waals and Coulomb solid–liquid interactions simultaneously. We tried three approaches: modifying solid–liquid L-J interactions and Coulombic interactions via λ = λ_LJ_ = λ_C_, (L-J/Coulomb scaling integration path; λ_LJ_λ_C_ path), modifying them via λ = λ_LJ_ = λ_cut(C)_, (L-J scaling/Coulomb cutoff integration path; λ_LJ_*r*_cut(C)_ path), and finally via λ = λ_LJ_ = λ_α_, (L-J scaling/Coulomb damping integration path; λ_LJ_α path), where the sampling range for all was 0 < λ ≤ 1. The work of adhesion of these integration paths closely resembles eqs 10–13:
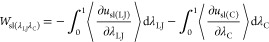
14
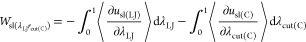
15
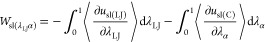
16

Because the work of solid–liquid adhesion of the same system must not depend on the integration path, all approaches should give identical values, except for numerical errors:
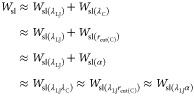
17

As in our previous work,^37^ the LAMMPS MD package^48^ was used to perform all simulations and part of the postprocessing with the integration being done by the velocity Verlet algorithm at a time step of 0.5 fs. The integration time step was chosen according to a general rule of using one-tenth of the fastest motion period, the vibration of the O–H water bond at approximately 9 fs.^49^

To obtain systems with different *r*_cut(C)_ and α for only solid–liquid interaction as described in eq 3, the *pair*_*style hybrid* capability was used. On the other hand, to implement different λ_C_, we had to use the *pair*_*style table* capability due to technical limitations, although this would not have been necessary if we used one of the available soft core Coulomb potentials. Finally, to efficiently compute Hamiltonian derivatives, new pair potentials matching the analytical Hamiltonian derivatives were constructed via the *pair*_*style python* capability and were then tabulated for *pair*_*style table* via *pair*_*write* command, using the bitmap style with 2^16^ points, with the inner and outer cutoff being set to 0.4 and 12 Å, respectively. The number of tabulation points was chosen by setting a high enough value to give identical results within numerical error. Once the tabulation was completed, the *rerun* capability was used to compute the Hamiltonian derivative values from simulation trajectory files.

### Numerical Integration Scheme

To obtain the work of adhesion values described in the previous section, we had to numerically integrate the mean Hamiltonian derivatives. One of the challenges of numerical integration is correctly estimating the error. In particular, for MD, computation of each data point is numerically expensive, so a good error estimation scheme is crucial to allow efficient sampling. Although many numerical integration schemes allow for theoretical error estimation, this requires higher derivatives of the integrand, which are usually unknown and numerical derivation is not realistic because of numerical instability.^50^

We, therefore, chose a simple adaptive quadrature algorithm, that is, an iterative algorithm that chooses new sampling points based on estimated local error in separate integration intervals, where the errors were estimated using a simple heuristic, similar to that in previous literature.^51^ We will describe the approach briefly here, and will refer to a χ integration path (χ path) when an operation is applicable to all of the integration paths. At step 1, λ is sampled at 65 equally spaced points in the range of [0, 1] for λ_C_, *r*_cut(C)_ and α paths. On the other hand, because of technical limitations, for λ_LJ_, λ_LJ_λ_C_, λ_LJ_*r*_cut(C)_, and λ_LJ_α paths, 64 equally spaced point in the range of (0, 1] are sampled and values at λ = 0 are obtained via extrapolation from the two nearest data points. At step 2, the work of adhesion for the χ path is obtained via both the trapezoidal rule and the definite integral of a fitted cubic B-spline curve as *W*_sl_^*t*^ and *W*_sl_^*s*^, respectively. B-spline fitting and integration is done via the SciPy library,^52^ which uses FITPACK for spline-related algorithms.^53^ These two integration schemes are chosen because of the simplicity and ease of accessibility, although other integration schemes can be used instead, provided the level precision is substantially different. At step 3, the global relative error is estimated as
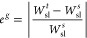
18and the local relative error per unit measure is computed for each interval between adjacent sampling points as
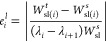
19where λ_*i*_ and λ_*i*+1_ are the sampling points of interval *i*, while *W*_sl(*i*)_^*t*^ and *W*_sl(*i*)_^*s*^ are the definite integrals over the interval *i* via the trapezoidal rule and fitted cubic B-spline, respectively. Note that for *W*_sl(*i*)_^*s*^ all the sampling points are used for the fitting. It is assumed that the relative error between the two estimates of different quadrature degrees, degree 1 for trapezoidal rule and degree 3 for B-spline integration, is at the same magnitude as the relative error between the more precise numerical integration scheme and the true value.^51^ While, in general, there is no such guarantee, for short intervals where the integrand is well behaved and its values do not change greatly, it has been demonstrated to hold and has been successfully used in various adaptive quadrature algorithms.^51^ The local relative error *per unit measure* was used instead of the simple local relative error to give larger weights to intervals where the integrand has high gradients, that is, steep peaks. If the global error *e*^*g*^ is smaller than the global error threshold *e*^*t*(*g*)^, and local errors for all integrals *e*_*i*_^*l*^ are smaller than the local error threshold *e*^*t*(*l*)^, the numerical integration is terminated, and *W*_sl_^*s*^ is used as the estimated value. Otherwise, the algorithm advances to the next step. At step 4, the *n* intervals with the largest errors are chosen, and new sampling points are picked at their midpoints. In principle, setting *n* = 1 would allow to reach the desired global error with the least additional sampling points, but it is beneficial to set it to a larger value as it allows parallel evaluation of partial Hamiltonian derivatives via MD simulation. In this work, all intervals with an error above a certain threshold were selected. The threshold was initially set to *e*^*t*(*l*)^, and then decreased by 0.01 in case of no intervals above it. The algorithm continues to step 2 afterward, unit both the global and local error estimates are below their thresholds.

The global relative error threshold was set to *e*^*t*(*g*)^ = 0.01, while the threshold of local relative error per unit measure was set to *e*^*t*(*l*)^ = 0.05. As briefly described in the section about the simulation system, at fixed λ the Hamiltonian derivatives were analytically computed via MD, and each point was sampled for 8 ns. To check the validity of the algorithm, Laguerre polynomials were also integrated to verify that the estimated error is of acceptable accuracy. Laguerre polynomials were chosen instead of, for example, Chebyshev polynomials used in previous literature,^51^ as Laguerre polynomials oscillate at varying periods, and their definite integrals are small when compared to the oscillation amplitudes, which is similar to worst-case scenarios for Hamiltonian derivatives observed in this work. The polynomial degrees were varied from 3 to 99, and the integration ranges were set from 0 to 1–20, with an increment of 1. In most cases good estimates were achieved, where the real relative error differed from the estimated one by less than 0.01. Only when the oscillation period became too short for the initial sampling to capture, did the error estimates become unreliable. For the Hamiltonian derivatives used in this work, even the worse-case ones had only few large oscillations that were fully captured by the initial sampling.

## Results and Discussion

### Solid–Liquid Potential Energies and Hamiltonian Derivatives

**Figure 2:**
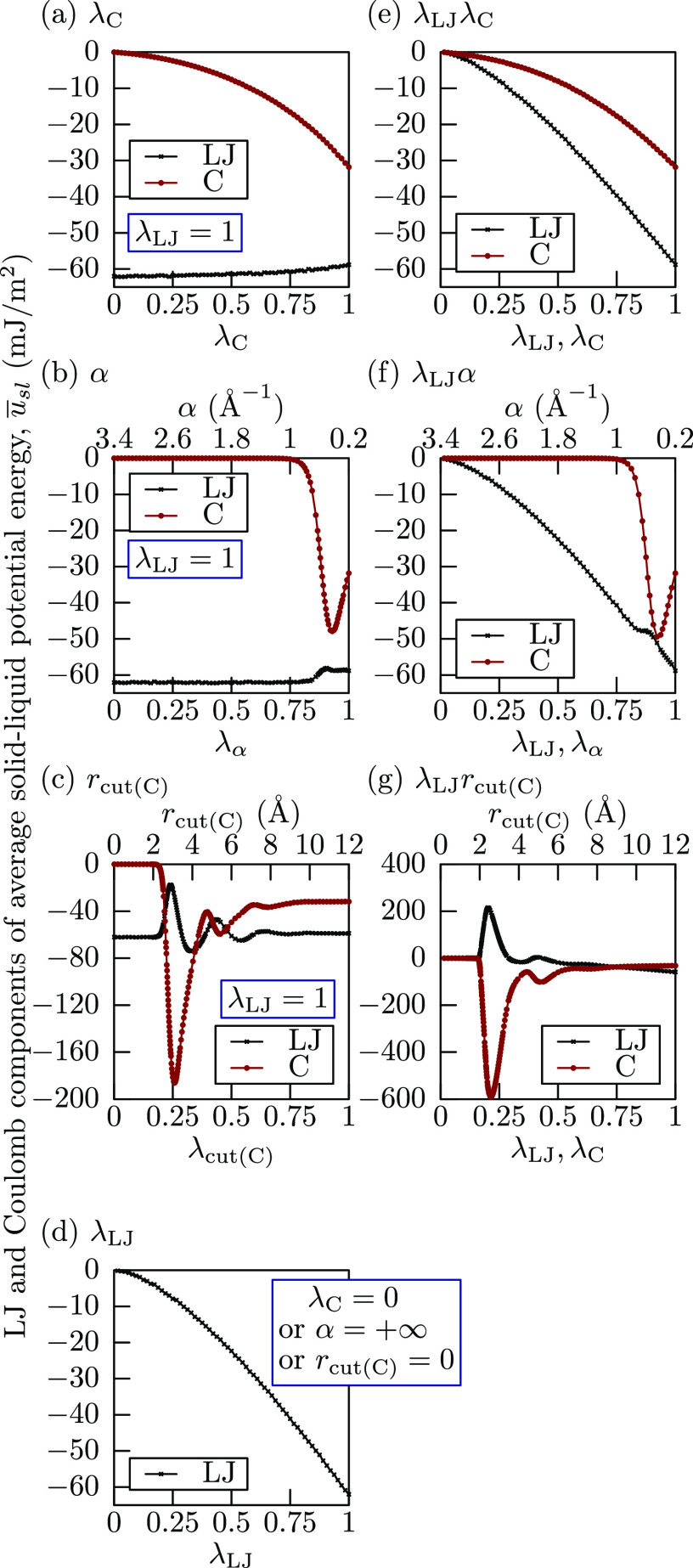
Solid–liquid energies for each integration path. The statistical error of the mean for each point was evaluated by block averaging^54^ and is at most 0.7 mJ/m^2^ for Coulomb cutoff integration paths (c, g), and at most 0.15 mJ/m^2^ for the remaining paths, and not show in the figure because of the small magnitude.

**Figure 3:**
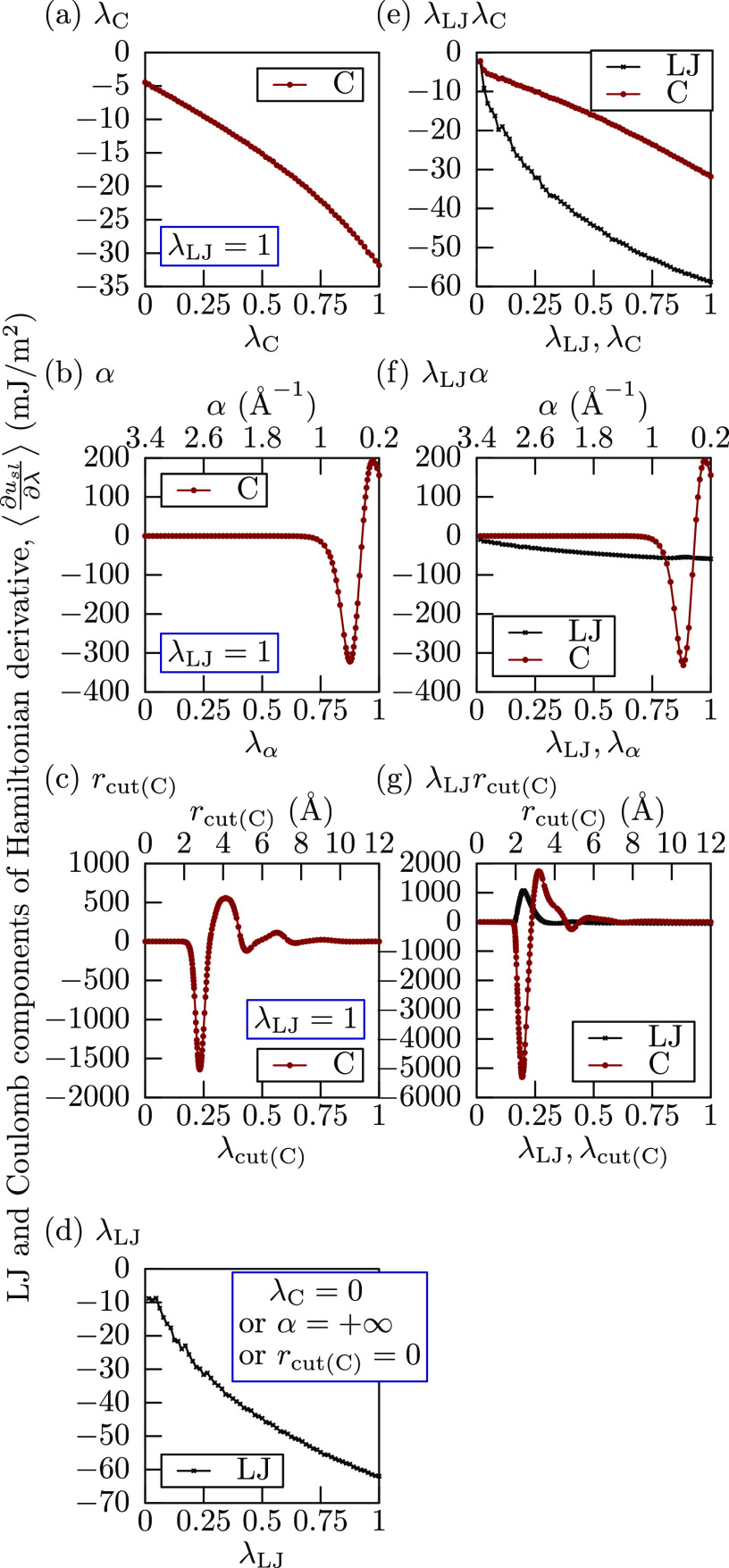
Hamiltonian derivatives for each integration path. The statistical error of the mean for each point was evaluated by block averaging^54^ and is at most 0.15 mJ/m^2^ for scaling-only integration paths (a, d, e), at most 0.9 mJ/m^2^ for Coulomb damping paths (b, f), and at most 8 mJ/m^2^ for Coulomb cutoff paths (c, g) and not show in the figure due to small magnitude.

Solid–liquid potential energies for all integration paths are displayed in Figure 2, where both L-J and Coulomb contributions are shown if applicable. Corresponding Hamiltonian derivatives are shown in Figure 3. As expected, all three Coulomb integration paths (λ_C_, α, and *r*_cut(C)_) in Figure 2a–c have identical L-J potential energies at λ = 0, while for other integration paths (λ_LJ_, λ_LJ_λ_C_, λ_LJ_α, and λ_LJ_*r*_cut(C)_) in Figure 2d–g the total potential becomes almost zero at λ ≈ 0.

The scaling integration paths (λ_C_, λ_LJ_, and λ_LJ_λ_C_) all show monotonic change in both potential energies in Figure 2a, d, e and Hamiltonian derivatives in Figure 3a, d, e, which is a highly desirable property from the point of numerically integrating. Integration paths with damping (α and λ_LJ_α) show a single potential energy dip with a local minimum in Figure 2b, f and a more pronounced dip/peak set in Hamiltonian derivatives in Figure 3b, f, which is slightly less desirable than a strictly monotonic change, but the total magnitude of the values is comparable to that in the scaling integration paths, and therefore should not be much more difficult to handle. On the other hand, integration paths with cutoff modification (*r*_cut(C)_ and λ_LJ_*r*_cut(C)_) show large potential energy oscillations in Figure 2c, g and even larger Hamiltonian derivative oscillations in Figure 3c, g. This is caused by shortened Coulomb cutoff, and such artifacts are reported in literature.^55^ Because of the large oscillation magnitude in comparison to the definite integrals, it is clear that these paths are the most difficult to evaluate correctly.

### Work of Solid–Liquid Adhesion

By integrating the values in Figure 3 according to eqs 10–17, we obtained the work of solid–liquid adhesion for a total of 6 integration paths, which is shown in Table 1. Also show in Table 1 is the result of a control silica–water system with long-range electrostatics, obtained via the λ_LJ_λ_C_ integration path with 8 sampling points, which provide sufficient precision, as will be described in more detail in the next section. For integration paths with both L-J scaling and Coulomb modification, L-J and Coulomb contributions of *W*_sl(LJ)_ and *W*_sl(C)_ from eqs 14–16 are also included. To adequately evaluate the precision of the computations and to make the source of possible inaccuracy clearer, the standard error of the mean due to statistical uncertainty is indicated in brackets, while the estimated error due to numerical integration is indicated after the ± symbol. To account for statistical inefficiency,^54^ the standard error of the mean was computed by dividing the sampled data into four blocks and individually conducting the numerical integration. The numerical error estimate was obtained in a manner equivalent to the relative global error estimate in eq 18. In case of α, λ_C_, and *r*_cut(C)_ paths, total work of adhesion *W*_sl_ was obtained by combining with λ_LJ_ path results, as indicated in eq 17, and numerical uncertainty and the numerical estimation were computed via error propagation.

**Table 1:** Work of Solid–Liquid Adhesion and Its L-J and Coulomb Components in Silica–Water Systems^a^

	*W*_sl(LJ)_	*W*_sl(C)_	*W*_sl_
λ_LJ_	41.57(6) ± 0.0007	N/A	N/A
λ_C_	N/A	16.10(1) ± 0.0002	57.67(6) ± 0.0007
α	N/A	16.12(1) ± 0.05	57.68(6) ± 0.05
*r*_cut(C)_	N/A	16.05(6) ± 0.08	57.62(8) ± 0.08
λ_LJ_λ_C_	40.95(8) ± 0.007	16.82(2) ± 0.0008	57.8(1) ± 0.008
λ_LJ_α	41.53(3) ± 0.001	16.76(2) ± 0.09	58.29(3) ± 0.09
λ_LJ_*r*_cut(C)_	–31.95(3) ± 0.03	90.7(1) ± 0.02	58.7(1) ± 0.008
λ_LJ_λ_C_ (PPPM)	55.55(5) ± 0.05	–4.69(1) ± 0.004	50.86(4) ± 0.04

a In case of α, λ_C_, and *r*_cut(C)_ paths, *W*_sl_ was obtained by combining with λ_LJ_ path results, as indicated in eq 17. The brackets indicate the standard error of the mean because of the statistical uncertainty in MD, while the values after the ± symbol are the estimated numerical integration error. The control system with long-range electrostatic interactions is indicated by “PPPM”. All values are in mJ/m^2^.

First, all integration paths for systems with damped Coulombic interactions gave comparable work of solid–liquid adhesion *W*_sl_ values of approximately 58 mJ/m^2^, which is close to 50.86 mJ/m^2^ of the control system with long-range electrostatics. It is also close to 48 mJ/m^2^ from our previous work,^37^ where we used integration paths equivalent to λ_LJ_ and λ_C_ paths, and the system is mostly identical with our control system with long-range Coulombic interactions, except the silica surface had slightly larger lattice spacing than in this work. The difference in the total work of adhesion between systems with damped and full, long-range electrostatics is quite significant and indicates that there can only be a semiquantitative match at most. This is not unexpected, as this family of damped interactions, originally derived by Wolf et al.,^39^ assumes a homogeneous bulk environment, although its applicability to systems with interfaces has also been investigated.^56,57^ To gauge the effect of damping in systems with stronger solid–liquid Coulomb interactions, the work of adhesion was also obtained for MgO–water systems via the λ_LJ_λ_C_ integration path with 8 sampling points, provided in Table 2. While, because of the ionic nature of the MgO crystal, the work of adhesion is more than twice that in silica–water systems, the difference between damped and undamped systems is also roughly 10 mJ/m^2^, which is very close to the silica–water systems. A stronger orientation of water hydrogen atoms toward the solid surface was observed in systems with damped Coulomb interactions, shown in Figure S2. This makes it difficult to devise an analytical correction, for example using a dielectric continuum model as in the Born hydration energy equation.^58,59^ In addition, the Wolf model has also been reported to underestimate the liquid–vapor interfacial tension.^56^ Both of these points are believed to contribute to a larger work of adhesion in systems with damped electrostatics. Finally, from the Hamiltonian derivative graph of the α path in Figure 3b, it appears that the Coulomb contribution to the work of adhesion could be reduced by decreasing the damping coefficient α, therefore, in theory, it should be possible to align the work of adhesion between the damped and long-range electrostatic systems exactly. The validity and soundness of doing so, however, is beyond the scope of this work.

**Table 2:** Work of Solid–Liquid Adhesion and Its L-J and Coulomb Components in MgO–Water Systems^a^

	*W*_sl(LJ)_	*W*_sl(C)_	*W*_sl_
λ_LJ_λ_C_	–11.70(3) ± 0.3	140.7(3) ± 0.4	129.0(3) ± 0.2
λ_LJ_λ_C_ (PPPM)	0.52(9) ± 0.3	117.4(2) ± 0.4	117.9(3) ± 0.1

a The brackets indicate the standard error of the mean due to statistical uncertainty in MD, while the values after the ± symbol are the estimated numerical integration error. System with long-range electrostatic interactions is indicated by “PPPM”. All values are in mJ/m^2^.

As a final note, it is worth pointing out the large difference between the L-J and Coulomb components, *W*_sl(LJ)_ and *W*_sl(C)_, for several of the integration paths in Table 1. As discussed in existing literature,^60^ even though we can unambiguously separate the L-J and Coulomb potential energies in our model, the same does not hold for their contributions to the work of adhesion, even though it is still a rather straightforward operation, as shown in eqs 10–16. Indeed, as the λ_LJ_λ_C_ path demonstrates, even qualitative tendencies are not guaranteed to be reproduced as the components are somewhat arbitrary and depend on the chosen integration path.

### Numerical Efficiency of Different Integration Paths

As can be observed from Table 1, even in the worst cases the error estimates of numerical integration are comparable to statistical uncertainty inherent in MD and they are all relatively small due to the adaptive quadrature algorithm we applied. This is true even for the cutoff integration paths (*r*_cut(C)_ and λ_LJ_*r*_cut(C)_), which resulted in problematic integrand shapes with large oscillations as shown in Figure 3c, g. Therefore, provided any integration path eventually gives the correct answer, it is preferable to select the one which needs the least sampling points.

**Figure 4:**
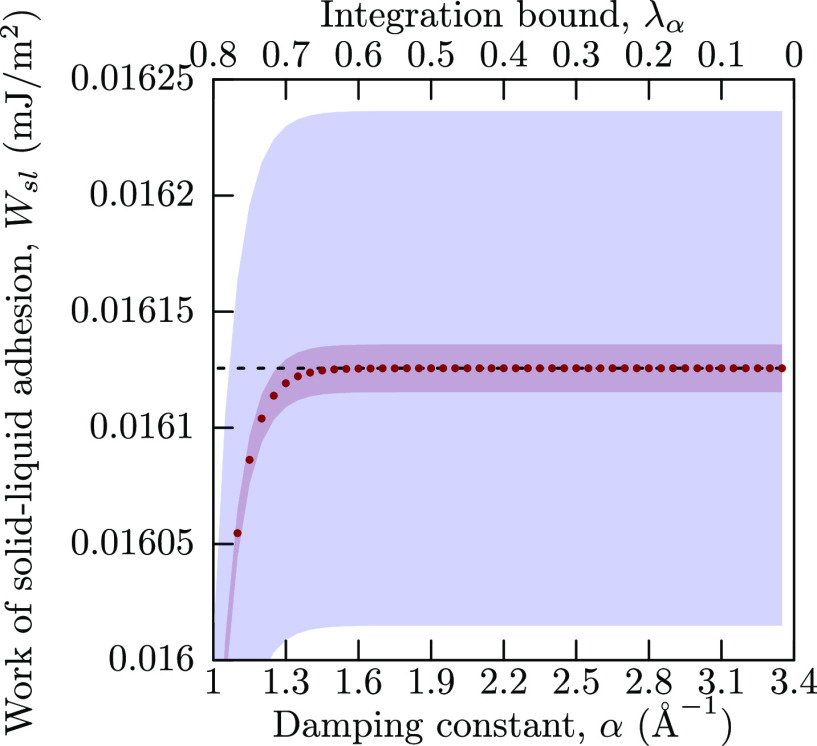
Cumulative work of solid–liquid adhesion from α path, obtained by changing the integration range. Inner filled red curves show the standard error of the mean because of the numerical uncertainty, while outer filled blue curves show the estimated numerical integration error. The dashed line is the value when integrated over the whole interval.

Before doing any further comparison between different integration paths, we note that for paths with modified damping (α and λ_LJ_λ_α_) in Figure 3b, f, the upper limit of the damping coefficient at 3.4 Å^–1^ appears to be too large, as relevant Hamiltonian derivatives are effectively zero already at α = 1.8 Å^–1^. This is demonstrated in Figure 4, where the cumulative work of adhesion, obtained by eq 12, is shown for increasing α, with the corresponding λ_α_ also indicated. From there, we can observe that an upper bound of α = 1.6 Å^–1^ (λ_α_ = 0.6) is enough for the cumulative value to converge within the margin of error. Thus, we will use this value when discussing numerical efficiency of the integration paths below. On the other hand, the same approach cannot be used with current data for the λ_LJ_α path, as the damping coefficient α is coupled with L-J interaction scaling parameter λ_LJ_.

Because of our adaptive quadrature algorithm, the exact number of iterations and the order of new sampling points highly depends on the number of new sample points chosen at each iteration, step 4 of the numerical integration scheme. Therefore, we chose to evaluate the error and precision based on the number of sampling points and not iterations. Numerical integration was redone, where only the sampling points at 2^*n*^ equally spaced intervals in the integration range were chosen. This was redone for each integration path with *n* starting from 3 to up to 12 for the λ_cut(C)_ path. Only for the α path, sampling points above α = 1.6 were omitted. Note that at *n* higher than 6, the total number of sampling points was lower than 2^*n*^ + 1 and the actual sampling order did not occur in the other of increasing *n*, both because of the adaptive quadrature algorithm.

**Figure 5:**
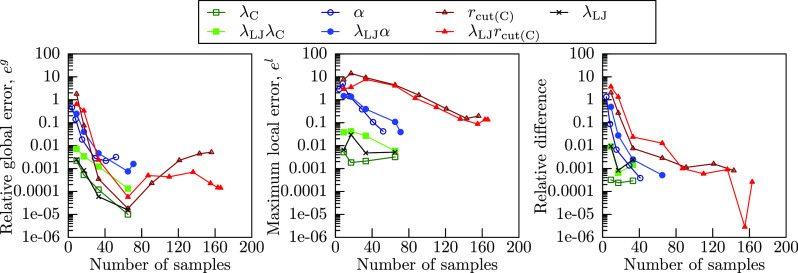
(Left) Estimated relative global numerical error of partial Hamiltonian numerical integration versus the number of sampling points. (Middle) Estimate of the maximum local relative numerical error per unit length of partial Hamiltonian numerical integration versus the number of sampling points. (Right) The relative difference from the values of the numerical integration of partial Hamiltonian with the most sampling points.

The relation of sampling points and precision is shown in Figure 5. The left panel of Figure 5 indicates the estimated global error of numerical integration, the center panel indicates the maximum value of the estimated local error per unit length of each integration interval, and the right panel shows the relative difference from the integral with the most sampling points. Note that due to the nature of logarithmic scale, data points for most sampling points are not shown in the right panel of Figure 5. From an overall point of view, the relative global error estimate of numerical integration roughly reflects the actual relative difference between the integral with the most sampling points. On the other hand, the maximum local error in the center panel shows substantially higher values than global error, and does not appear to converge even at a large sampling point number.

First, looking at the left panel of Figure 5, we notice a sudden increase in numerical error after 29 sampling points for the α path and 65 points for *r*_cut(C)_, λ_LJ_α, and λ_LJ_*r*_cut(C)_ paths. This is the point where adaptive quadrature algorithm started and the integration intervals were no longer of equal length. It has been demonstrated that the trapezoidal rule is very efficient at integrating peak functions,^61^ and a switch to a nonequally spaced sampling could indeed decrease the precision of trapezoidal integration, increasing the estimated numerical error. This, however, does not mean that the precision of the spline integral was decreased. It is interesting to note that as far as the relative global error estimate for numerical integration *e*^*g*^ is concerned, it was below the threshold of *e*^*t*(*g*)^ = 0.01 for all integration paths after the initial sampling and the algorithm could have been terminated if that had been the only criterion. Because the maximum relative local error estimate *e*_*i*_^*l*^, shown in the center panel of Figure 5, was above the threshold of *e*^*t*(*l*)^ = 0.05 for α, *r*_cut(C)_, λ_LJ_α, and λ_LJ_*r*_cut(C)_ paths, the adaptive quadrature algorithm turned on and increased the number of sampling points. On the other hand, the scaling-only integration paths (λ_LJ_, λ_C_, and λ_LJ_λ_C_), had both the global and local error estimates below their thresholds, and finished without applying the adaptive quadrature algorithm. In fact, the Hamiltonian derivatives (Figure 3a, d, e) for these integration paths are very well behaved, and even with as few as 9 sampling points, their relative global estimate is below the threshold. This is not just an artifact or underestimation, as in the left panel of Figure 5, we can observe that the relative difference between the numerical integrals with most sampling points is below 0.01. Therefore, we can conclude that the integration paths with only scaling (λ_LJ_, λ_C_, and λ_LJ_λ_C_) are the most numerically efficient. From the point of fewest sampling points, the λ_LJ_λ_C_ path is the most efficient, as the scaling is done at once for both L-J and Coulomb interactions. The second most efficient integration path is the α path, as even though it has a dip and a peak in the Hamiltonian derivative (Figure 3b) and needed the adaptive quadrature algorithm, the overall number of sampling points is relatively low, due to only needing to sample in the range of 0.2 Å^–1^ ≤ α ≤ 1.6 Å^–1^. This should be also applicable to the λ_LJ_α path, provided we choose this integration range from the start and adjust the λ_LJ_ and α relation. Coulomb cutoff integration paths (*r*_cut(C)_ and λ_LJ_*r*_cut(C)_) proved to be most difficult and inefficient to numerically integrate due to their steep peaks and dips in Figure 3c, g, and they needed the most iterations to reach a required precision. In fact, the relative local error *e*_*i*_^*l*^ of *r*_cut(C)_ and λ_LJ_*r*_cut(C)_ paths never converged to below the threshold of *e*^*t*(*l*)^ = 0.05. Iterations were in fact terminated after the relative global error was small enough. Overall, modifying the Coulomb cutoff to obtain the work of adhesion should be avoided if possible from the point of numerical efficiency. Although our choice prioritized steep peaks, as intended, the local error formulation in eq 19 also had the problem that it was slow to converge (center panel of Figure 5). This might be due to integration intervals approaching 0, as been briefly mentioned by Gonnet in his review;^51^ therefore, the local error might never converge. From an algorithmic point of view, this is suboptimal. Hence either only the global error estimate should be used for the algorithm termination, as we eventually did with *r*_cut(C)_ and λ_LJ_*r*_cut(C)_ paths or an alternative local error formulation is needed, which would properly decrease to 0 when the integration interval approaches 0, although it might be still preferable to have it prioritize steep peaks.

## Conclusion

In this work, we investigated how to efficiently obtain the solid–liquid work of adhesion from MD systems with damped Coulomb interactions by using the dry-surface approach,^28^ which is a thermodynamic integration method. This was done to demonstrate an alternative to using systems with long-range Coulombic solid–liquid interactions because modifying only solid–liquid interactions is nontrivial in practice, as it would require solving additional Poisson equations, which is not available in most MD software packages. This has been circumvented by fixing solid surface atoms in place in previous works,^37^ but this is also often undesirable. For damped Coulomb interactions, the formulation by Fennell et al.^38^ was used, as it let us to both modify the damping coefficient and cutoff length, while maintaining a smooth potential curve at the cutoff, but the conclusions should also be applicable to other formulations, such as soft core potentials.

A total of 6 different reversible paths were chosen for thermodynamic integration, with either scaling the solid–liquid Coulomb interactions, modifying their damping coefficient or changing their Coulomb cutoff length, and also scaling their Lennard-Jones (L-J) interactions either at the same time or separately from the Coulomb ones. Regardless of the integration path, the same work of solid–liquid adhesion values were obtained but differed by a non-negligible amount (approximately 10 mJ/m^2^) from that of systems with long-range Coulomb interactions, although the discrepancy was mostly unchanged regardless if the Coulombic interactions over the solid–liquid interface were weak or strong. Because of the different liquid structure near the interface being different for the two treatments, an analytical correction might be not trivial. It might be possible, however, to obtain close-enough values by increasing the damping coefficient, although further validation has not been done. As it currently stands, although the approach presented in this work does not produce the work of solid–liquid adhesion that is quantitatively comparable to that obtained from systems with long-range Coulombic interactions, the discrepancy appears to be mostly unchanged across wide range of solid–liquid interaction strengths, akin to a sort of systematic error. Therefore, applying the dry-surface method to systems with damped Coulomb interactions still allows qualitative comparison between different interfaces, with an added benefit of a much lower computational cost. The comparison should be especially efficient if the two interfaces are similar, such as having different functional groups, which would allow qualitative comparison by determining the discrepancy using a control system.

Even though all integration paths resulted in the same work of adhesion values due to being reversible, different numbers of sampling points were needed to obtain an acceptable precision because numerical integration was used. To increase the efficiency of data sampling for more difficult paths, an adaptive quadrature algorithm was successfully used. The fewest sampling points were required for integration paths with simple interaction scaling. More sampling points were needed for integration paths with changing of the damping coefficient, and most were needed when the Coulomb cutoff was modified, due to large potential energy oscillations at shorter cutoff values. Therefore, interaction scaling should be used when available, with damping coefficient modification also being acceptable, while the cutoff modification approach should be avoided if possible.

From the point of the equations in the process of obtaining the work of adhesion, it is also possible to split free energy contributions to various components, such as L-J and Coulomb. However, as is known from thermodynamics, while the total work of adhesion does not depend on the integration path, the components can vary greatly and are path dependent. Therefore, it is not possible to divide free energy into separate contributions unambiguously.

## Supporting Information Available

Illustration of MgO–water simulation systems and number density distributions of oxygen and hydrogen atoms in water at the solid–liquid interfaces (PDF)
