# Supplementary material for: Computing the Work of Solid–Liquid Adhesion in Systems with Damped Coulomb Interactions via Molecular Dynamics: Approaches and Insights
Source: J Phys Chem A. 2022 Aug 5;126(32):5506–16. doi: 10.1021/acs.jpca.2c03934 (PMC9393893; doi:10.1021/acs.jpca.2c03934)
Supplement: Supplementary file 1 — jp2c03934_si_001.pdf [file jp2c03934_si_001.pdf]

Computing the Work of Solid-Liquid Adhesion in Systems with Damped Coulomb Interactions via Molecular Dynamics: Approaches and Insights

Donatas Surblys,*,† Florian Müller-Plathe,‡ and Taku Ohara†

†*Institute of Fluid Science, Tohoku University, 2-1-1 Katahira, Aoba-ku, Sendai, 980-8577, Japan*

‡*Eduard-Zintl-Institut für Anorganische und Physikalische Chemie, Technische Universität Darmstadt, D-64287, Germany*

E-mail: donatas@tohoku.ac.jp

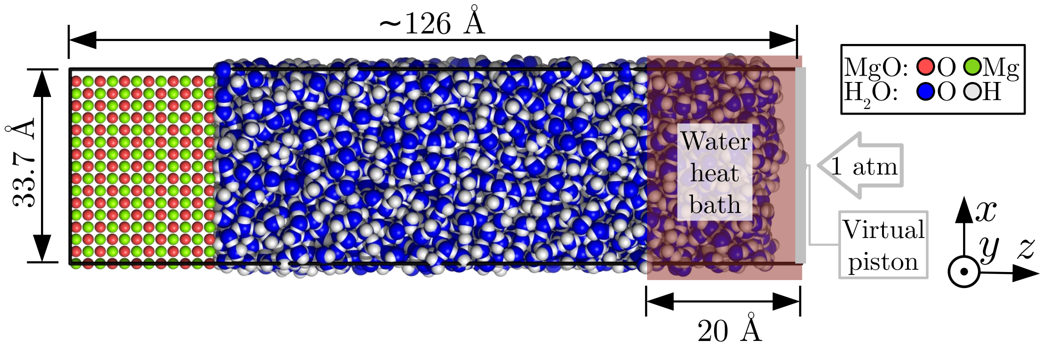

Figure S1: Front view of the MgO-water simulation system. The depth (y) dimension is 33.7 Å.

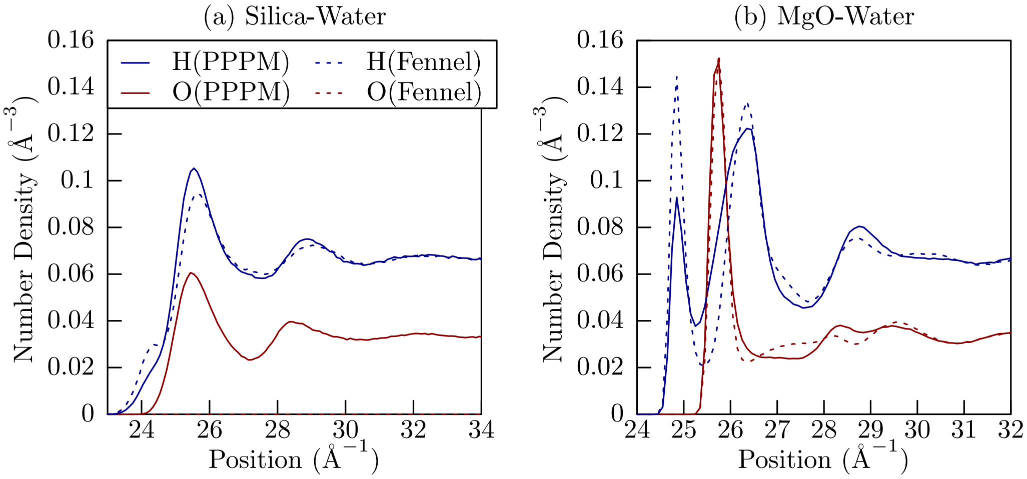

Figure S2: Number density of water hydrogen and oxygen atoms at the solid-liquid interface in (a) silica-water and (b) MgO-water systems under full long-range Coulomb interactions (PPPM) and dampled Coulomb interactions (Fennel).
